# Optimized Radio Frequency Footprint Identification Based on UAV Telemetry Radios

**DOI:** 10.3390/s24165099

**Published:** 2024-08-06

**Authors:** Yuan Tian, Hong Wen, Jiaxin Zhou, Zhiqiang Duan, Tao Li

**Affiliations:** 1 College of Aeronautics and Astronautics, University of Electronic Science and Technology of China, Chengdu 611731, China; ocotea@163.com; 2 School of Unmanned Aerial Vehicle Industry, Chengdu Aeronautic Polytechnic, Chengdu 610100, China; zhoujiaxin1214@163.com (J.Z.); maizi_duan@163.com (Z.D.); li249856@163.com (T.L.)

**Keywords:** RF fingerprinting, UAV identification, telemetry radios, convolutional neural network, self-organizing map

## Abstract

With the widespread use of unmanned aerial vehicles (UAVs), the detection and identification of UAVs is a vital security issue for the safety of airspace and ground facilities in the no-fly zone. Telemetry radios are important wireless communication devices for UAVs, especially in UAVs beyond the visual line of sight (BVLOS) operating mode. This work focuses on the UAV identification approach using transient signals from UAV telemetry radios instead of the signals from UAV controllers that the former research work depended on. In our novel UAV Radio Frequency (RF) identification system framework based on telemetry radio signals, the EC−α algorithm is optimized to detect the starting point of the UAV transient signal and the detection accuracy at different signal-to-noise ratios (SNR) is evaluated. In the training stage, the Convolutional Neural Network (CNN) model is trained to extract features from raw I/Q data of the transient signals with different waveforms. Its architecture and hyperparameters are analyzed and optimized. In the identification stage, the extracted transient signals are clustered through the Self-Organizing Map (SOM) algorithm and the Clustering Signals Joint Identification (CSJI) algorithm is proposed to improve the accuracy of RF fingerprint identification. To evaluate the performance of our proposed approach, we design a testbed, including two UAVs as the flight platform, a Universal Software Radio Peripheral (USRP) as the receiver, and 20 telemetry radios with the same model as targets for identification. Indoor test results show that the optimized identification approach achieves an average accuracy of 92.3% at 30 dB. In comparison, the identification accuracy of SVM and KNN is 69.7% and 74.5%, respectively, at the same SNR condition. Extensive experiments are conducted outdoors to demonstrate the feasibility of this approach.

## 1. Introduction

Civil UAVs have been widely used in aerial photography, delivery systems, civil infrastructure inspection, and search and rescue operations with the development of UAV-related technologies and products [1]. Even though so many beneficial civil applications of UAVs, some news of UAVs violating public privacy and the security of sensitive facilities have been reported online. Therefore, there is an urgent need to ensure the safety of airspace and ground facilities in the no-fly zone [2]. Accurate detection and identification of unauthorized UAVs is an effective way to realize UAV flight supervision [3].

Several techniques such as radar detection, photoelectric detection, and sound detection have been proposed for UAV supervision and defense so far [4,5]. Radar is the most common mechanism for detecting UAVs. A radar system uses a radio transmitter to broadcast radio pulses toward the UAV so that the UAV can reflect the radio waves. A receiver receives the reflected waves and the time characteristics of the reflected radio waves can be used to identify the UAV [6]. Radar-based techniques mostly fail to detect micro-UAVs due to the small size, slow speed, and low altitude of UAVs. Photoelectric technology involves the use of video surveillance cameras to monitor restricted areas [7]. The detection capability of the optoelectronic detection system is limited by the environment and its field of view. If the UAV appears out of the field of view or is shielded in a complex environment, the system may not be able to detect the target in time. UAVs generate sounds when their propellers rotate. The sound waves propagated from a given UAV are adopted as the audio fingerprint of the UAV [8]. The sound detection system is only suitable for short-range scenarios due to ambient noise.

Some of these challenges can be solved by RF fingerprint identification techniques [9]. The UAV is usually controlled by a remote controller or Ground Control Station (GCS). The remote controller is a handheld device used to remotely pilot and control a UAV in manual control mode by RF signals. The current trend in RF fingerprint identification of UAVs focuses mainly on the RF signals between UAVs and remote controllers [10]. However, the remote controller’s power is often low, which is not suitable for long-distance signal detection and identification [11].

In fact, GCS serves as the primary interface between the operator and the UAV in autonomous flight mode, allowing for real-time control over its movements, navigation, and operation. The wireless datalink composed of the UAV telemetry radio and GCS telemetry radio is fundamental to guarantee the UAV’s safe operation. Compared with the remote controller, the wireless communication distance of the UAV telemetry radio is much larger. Therefore, UAV telemetry radio signals as signatures have a greater advantage.

The hardware imperfection of UAV telemetry radios can generate a unique fingerprint that is attached to the radio signals. The aim of this paper is to propose a detection and identification approach based on RF fingerprints of UAV telemetry radios. The transient signals of UAV telemetry radios are extracted first. Raw I/Q data of transient signals are used to train CNN models. We propose a CSJI algorithm to improve the accuracy of RF fingerprint identification. A testbed is constructed, which consists of two UAVs as the flight platform, a USRP as the receiver, and 20 telemetry radios as targets for identification. The performance of the proposed approach is evaluated indoors and outdoors with the testbed.

The main contributions of this paper can be summarized as follows:

- We study typical technologies for UAV RF fingerprint identification. We propose a UAV RF fingerprint identification framework based on telemetry radios in a scenario where multi-UAVs co-exist in the same frequency band;
- The EC−α algorithm is optimized to extract the transient signals of the UAV telemetry radios and the starting point detection accuracy at different SNRs is analyzed. Aiming at the telemetry radio’s characteristics of using multiple waveforms, the raw I/Q data of transient signals are used to train the CNN models. The paper investigates the identification performance influenced by CNN model parameters;
- To further improve the identification performance, we propose and implement a CSJI algorithm, which uses clustered transient signals and the CNN model to jointly identify the RF footprint of UAV telemetry radios. The performance of the CSJI algorithm is compared and analyzed with KNN and SVM at different SNRs;
- We conduct numerous indoor and outdoor experiments to evaluate the effectiveness of our approach. It demonstrates that the optimized identification approach achieves an average accuracy of 92.3% indoors at 30 dB and an average accuracy of 76.1% at around 25 dB outdoors.

The remainder of the paper is organized as follows. Section 2 gives an overview of the related work for UAV detection and classification; Section 3 describes the UAV RF identification system framework based on telemetry radio signals; Section 4 provides the proposed detection and identification techniques; and Section 5 introduces the experimental setup and presents the results. The performance evaluation and results are discussed. Section 6 provides the concluding remarks and future work.

## 2. Related Works

This section summarizes some recent research related to UAV detection and identification. With the development of machine learning and artificial intelligence, numerous machine learning algorithms and deep learning have been applied to identify UAV RF fingerprints in the literature [12,13]. To the best of our knowledge, there is no relevant research on RF fingerprint detection and identification based on UAV telemetry radio signals.

The signals transmitted from the controller were used to detect and classify the Micro-UAV. This method was robust to noise and the KNN algorithm was used to achieve an average classification accuracy of 96.3% [14] but this approach did not perform outdoor environmental impact analysis. An improved RF-based method to detect UAVs was proposed in [15]. Spectrum accumulation and statistical fingerprint analysis were employed to provide two frequency estimates. The recognition rate with this method was close to 100% within a distance of 2.4 km and greater than 90% within a distance of 3 km. However, this method can only determine if a UAV was present using an RF signal at 2.4 GHz. In [16], the authors designed a radio frequency signal classification toolbox, which can be used to detect and classify radio frequency signals of UAV communication systems, and discussed how to use software radio to realize UAV detection and classification. This method only used two different types of UAVs for testing. A software radio-based civil UAV radio signal detection, classification, and analysis system focusing on the application of machine learning technology was proposed in [17]. The concept has not been experimentally tested and evaluated. A method for detecting consumer-grade UAV signals was proposed by performing fingerprint statistical analysis on WiFi networks in [18]. This method did not provide sufficient analysis of the parameters of the machine learning algorithm and was tested with only 2 UAVs. The authors developed a CNN-based drone detection approach based on RF signals and provided a comparison with the state-of-the-art approach [19]. The CNN-based identification performance of this method was only tested on three UAVs. The authors performed drone signal detection, spectrum localization, and classification using two stages and combined detection and classification methods. The performance may deviate in the presence of unknown or newer UAV signals [20]. It was shown that the radio control signal sent to a UAV using a typical transmitter can be captured and analyzed to identify the controlling pilot using machine learning techniques. The classification accuracy depends on the flight trajectory [10]. In [21], the authors proposed a machine-learning RF-based DDI system that uses low-band RF signals from drone-to-flight controller communication. But this system did not discuss how interference can affect the feature property of the RF-signature spectrum.

Table 1 summarizes the relevant research work on UAV RF fingerprinting in terms of identification target types, preprocessing and detection methods, signal features, identification algorithms, number of test devices, and algorithm performance.

## 3. System Model Overview

**Figure 1:**
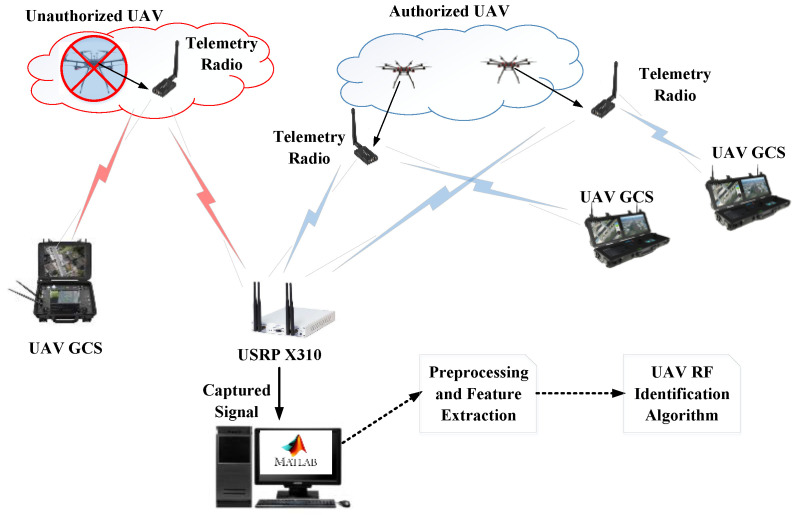
The scenario of the telemetry radio-based UAV identification system.

Most UAVs use telemetry radios as a datalink communication system while performing the mission. The uniqueness of the signature is inherently attributed to the electronic circuitries of the radio. The RF signals of a telemetry radio between the UAV and its GCS can be intercepted and collected as RF signatures for UAV identification. Figure 1 shows the scenario of the telemetry radio-based UAV identification system. In this scenario, there are two types of UAVs with different attributes. One is authorized and the other is unauthorized. All UAVs are controlled by GCSs via telemetry radios. The signals from telemetry radios are captured by USRP X310 (Ettus Research, Santa Clara, CA, USA) in a stealth manner. The RF signals serve as the signature to associated radios. Then, they are preprocessed and used to extract features. The extracted features are used to train an optimized CNN model for UAV identification.

**Figure 2:**
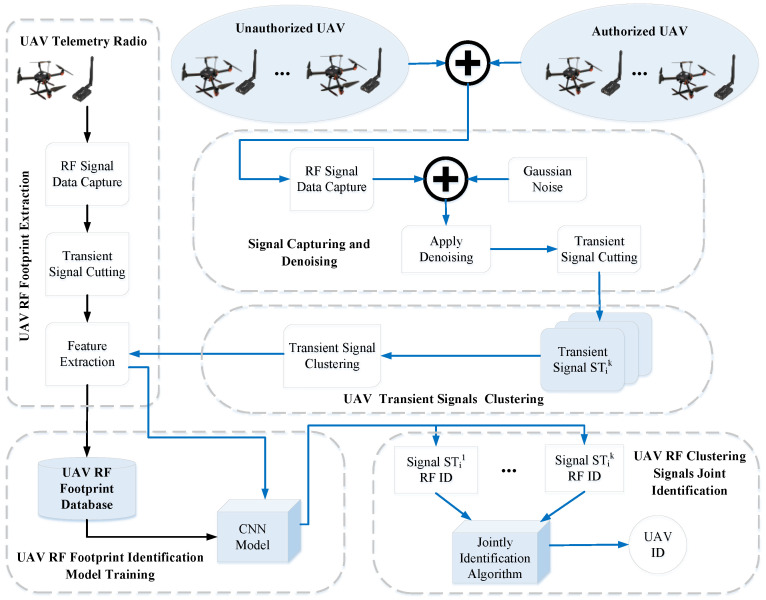
UAV RF identification system framework based on telemetry radio signals.

Our overall goal in this paper is to develop a framework capable of detecting and identifying a UAV in a scenario where multi-UAVs co-exist in the same frequency band. The system framework shown in Figure 2 gives an overview of our methodology. The framework is divided into two major stages. The first stage is establishing the RF fingerprint features database and RF fingerprint identification model. The second stage is applying and verifying RF fingerprint identification for authorized and unauthorized UAVs. This framework includes signal capturing, signal denoising, transient signal extraction, CNN training, signal clustering, and clustering signal joint identification.

## 4. System Implementation

### 4.1. UAV Transient Signal Detection

A UAV telemetry radio signal has two parts: the transient state and the steady state. The transient state occurs during power on and off of devices. It has a unique amplitude variation that makes the radio distinguishable. Therefore, transient signals of telemetry radio can be used to extract a unique signature for UAV identification. In our work, the UAV RF signals are sampled in a lab environment; therefore, the noise is stable for all measurements. To evaluate transient signal detection performance at the different SNRs, additive white Gaussian noise (AWGN) is added to the raw data by considering a range of SNR levels from 0 dB to 30 dB.

After signal sampling and noise adding are completed, the first step is detecting transient signals from the raw data. When there is no signal in the sampling frequency range, the raw data obtained are channel noise. The power begins to increase shortly after the start of the transient signal. To improve the efficiency of transient signal detection, the sampled raw data are cut into fixed-length data according to the time window and the variance of the data in each window is calculated first. The variance differential trajectory of the signal is obtained by the following formula:

(1) $$P_{diff}(i)=P_{x}(i+1)-P_{x}(i),\;i=1,2,\ldots ,L-1$$

(2) $$P_{x}(s)=\frac{1}{N_{w}}\sum_{i=1+(s-1)N_{w}}^{s\;N_{w}}{\left[x_{i}-\mu_{\omega (s)}\right]}^{2},\;s=1,2,\ldots ,L$$

x is the amplitude vector of sampled signal with noise, L is the maximum number of segments for signal cutting, $N_{w}$ is the length of the time window, and ${\mu_{\omega }}_{\left(s\right)}$ is the mean of the vector x within the $s_{th}$ window. $\delta_{T}$ is the variance differential trajectory detection threshold for each signal segment. If $P_{diff}(i)\geq \delta_{T}$, there is a starting point of the transient signal in this segment.

**Figure 3:**
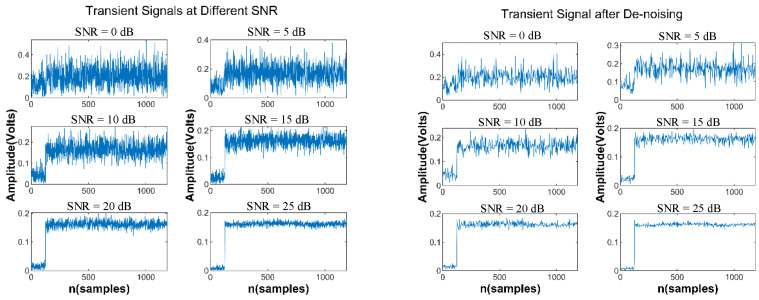
Transient signal de-noising.

The detected transient signal segments contain a significant amount of noise data. Processing it directly will decrease the accuracy of the starting point detection. De-noising is the most important part of preprocessing. The dB2 wavelet is used to decompose the signal into two levels for denoising. At each level, the raw signal data can be decomposed into a series of approximate and detail components by the wavelet de-noising method. Noise elimination can be achieved using a certain threshold of processing and wavelet reconstruction. See Figure 3.

The accuracy of starting point detection has a very important impact on the training of the UAV RF fingerprint identification model and signal identification test, especially in low SNR conditions.

An energy criterion (EC−α) is used to detect the signal starting point in this work. The idea underlying the EC−α is that the arrival of a radio signal is assumed to be characterized by a variation of its energy. The calculated energy curve ($E_{i}^{\prime }$) qualifies a global minimum that is considered as the arrival time of signals. The sample that corresponds to the global minimum is chosen as the starting point of transient signal. Compared with other detection algorithms, it yields superior performance in detection accuracy and computational complexity [27].

The energy ($E_{i}$) of a sampled signal (x) is defined as follows:

(3) $$E_{i}=\sum_{k=0}^{i}x_{k}^{2},\;i=1,\ldots ,N$$

where *N* is the signal length. The signal is separated from the noise part by:

(4) $$E_{i}^{\prime }=E_{i}-i\delta =\sum_{k=0}^{i}\left(x_{k}^{2}-i\delta \right)$$

where δ is a negative parameter and can be expressed as:

(5) $$\delta =\frac{E_{N}}{\vartheta \cdot N}$$

**Figure 4:**
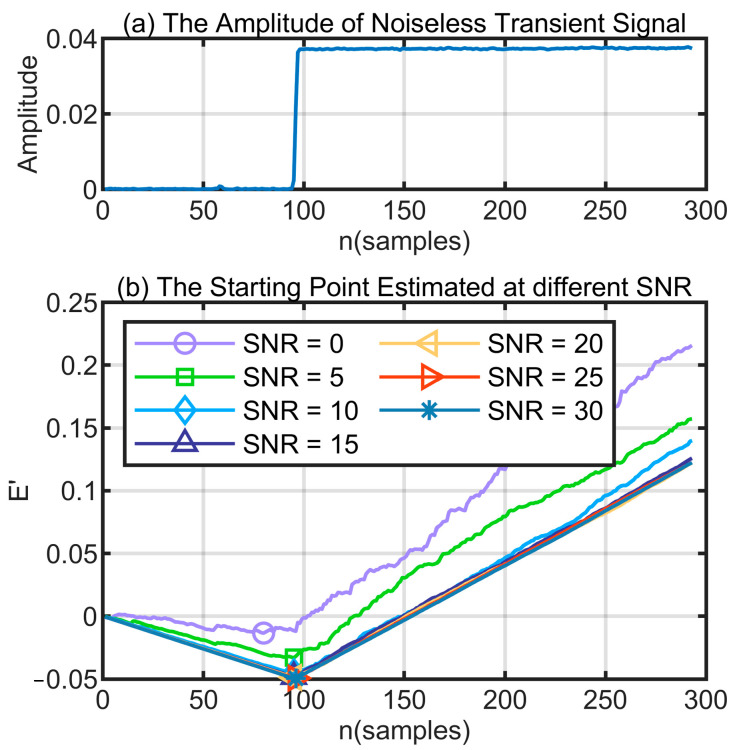
Transient signal cutting from the telemetry radio signal.

δ depends on the total energy of the signal ($E_{N}$) and the ϑ factor enables a reduction in the delaying effect of δ. As shown in Figure 4, the effect of starting point detection at different SNRs is demonstrated using the EC−α algorithm.

### 4.2. CNN Model Training Based on Raw I/Q Data

**Figure 5:**
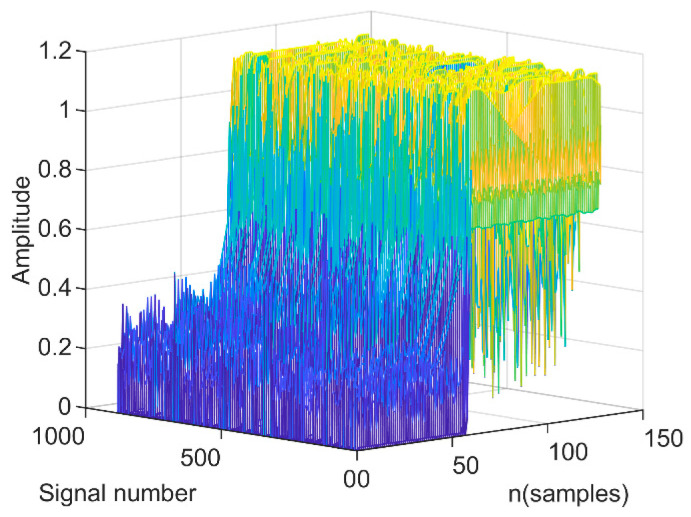
Different waveforms of the transient signals.

To the best of our knowledge, in the related research of radio frequency fingerprint identification based on transient signals, most of them use machine learning or artificial intelligence algorithms to identify transient signals with the same waveform. In our experiments, we find that there are many different types of waveforms used in the extracted transient signals of telemetry radios. Figure 5 shows the transient signal amplitude of a telemetry radio extracted during the sampling period.

Different types of transient signals have different waveform features in the time domain and frequency domain. The traditional identification algorithm based on a single type of waveform feature cannot meet the requirements of multi-type transient signal identification of UAV telemetry radios.

The Convolutional Neural Network based on raw I/Q samples has the superiority of combining feature extraction and identification into a model. The main advantage of this approach is that the hardware impairments of the transmitters embedded in the I/Q samples can be directly used to differentiate radios. Related references show that using features learned by CNN models to distinguish individual emitters is more reliable than manually designed features [28,29]. 

**Figure 6:**
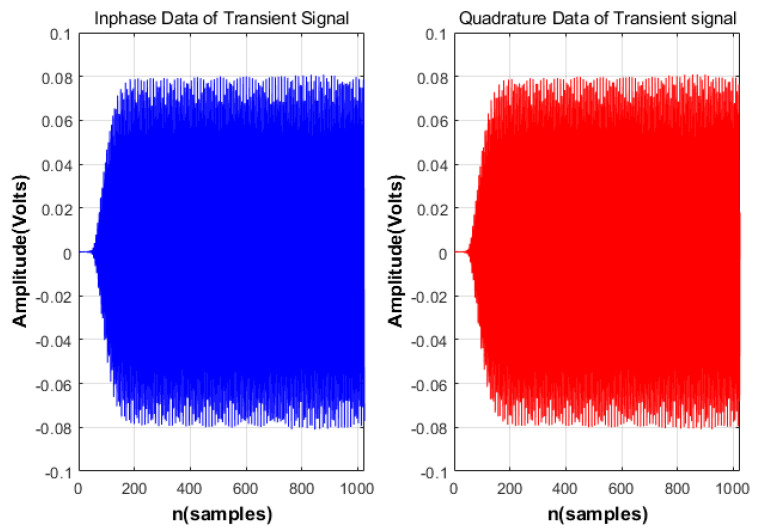
Raw I/Q samples of the telemetry radio.

The proposed baseline CNN model consists of two stages, namely, the training stage and the identification stage. In the former, a CNN is trained using raw I/Q samples of transient signals collected in the UAV transient signals detection section as shown in Figure 6. In the identification stage, raw I/Q samples of unknown telemetry radios are fed to a trained CNN and the telemetry radios are identified based on the observations at the output layer.

**Figure 7:**
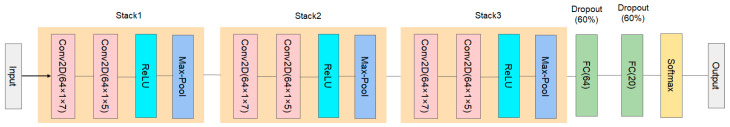
The architecture of the baseline CNN model.

Our baseline CNN depicted in Figure 7 is similar to the ORACLE architecture [30]. In this section, we first describe the CNN architecture and then introduce the input data requirement. The input to the CNN is a sequence of raw I/Q samples with a length of 128. Each complex datum is represented as two-dimensional real values, which results in the dimension of our input data growing to 2 × 128. This is fed to the first convolution layer. Convolutional layers are used to extract features by applying filters to the input data. It includes a set of filters that perform convolution operations on the input data. After the first convolutional layer, there is a second convolutional layer. The second convolutional layer is followed by the Rectified Linear Unit (ReLU). To reduce computational complexity and cost, the pooling layer applies downsampling techniques, which will greatly reduce the dimension of the data. An I/Q data feature extraction block is composed of two convolutional layers: one ReLU layer and one pooling layer. We stack three feature extraction blocks. There are two fully connected layers in the CNN architecture. They use the features extracted from the previous layers. To avoid the issue of overfitting, we added a dropout layer with a 60% dropout rate after each fully connected layer. In the output layer, a softmax activation function is used.

### 4.3. Clustering Signal Joint Identification Algorithm

The CNN model can achieve good identification performance in the RF fingerprinting of different types of devices. Since the telemetry radios in our experiments are the same model and they use different types of waveforms, their RF fingerprints are more complex and difficult to identify. So, it is a great challenge to identify transient signals through a CNN model in our work. To improve the performance of our approach, we propose a joint identification algorithm using clustering signals.

As shown in Figure 2, during each USRP RF signal data capture, many transient signals can be extracted from the raw data. However, it is difficult to determine a group of transient signals from the same device because there may be other similar radios in the sampling frequency range. In this paper, SOM is used to cluster transient signals, which come from the same telemetry radio. Then, raw I/Q data of transient signals clustered by SOM are fed to the trained CNN model. Each transient signal is identified through the trained CNN model. Finally, according to the identification result of each transient signal, the CSJI algorithm is used to identify the UAV telemetry radio.

The SOM algorithm is an unsupervised learning algorithm for clustering. It is a neural network with only an input layer and a hidden layer. A node in the hidden layer represents a class for clustering. Competitive learning methods are employed during the training process. Each input sample identifies its best-matching node in the hidden layer, referred to as its activation node, and subsequently updates the parameters of the activation node. Nodes adjacent to the active node will also appropriately update their parameters based on the distance from the active node. SOM does not require a predefined number of clusters. Additionally, SOM is less sensitive to the choice of initialization parameters.

The clustering operation is achieved by calculating the differences between all of the input vectors $x_{i},\;i=1,2,\ldots ,n$ and each weight vector $w_{j},\;j=1,2,\ldots ,n$, which combines the input vectors with the neuron j in the SOM. The similarity between $x_{i}$ and each neuron is usually calculated using the Euclidean distance

(6) $$d_{ij}=\sqrt{\sum_{k}{\left[x_{ik}-w_{jk}\right]}^{2}}$$

where k denotes the $k_{th}$ component of the vector. After the calculation, the neuron with the smallest distance is the winner. The winning neuron weight is calculated as follows:

(7) $$w_{j}(t+1)=w_{j}(t)+h_{cj}(t)\left(x_{i}(t)-w_{j}(t)\right)$$

where the $h_{cj}(t)$ is the neighborhood kernel centered on the winner unit and t denotes the time.

**Figure 8:**
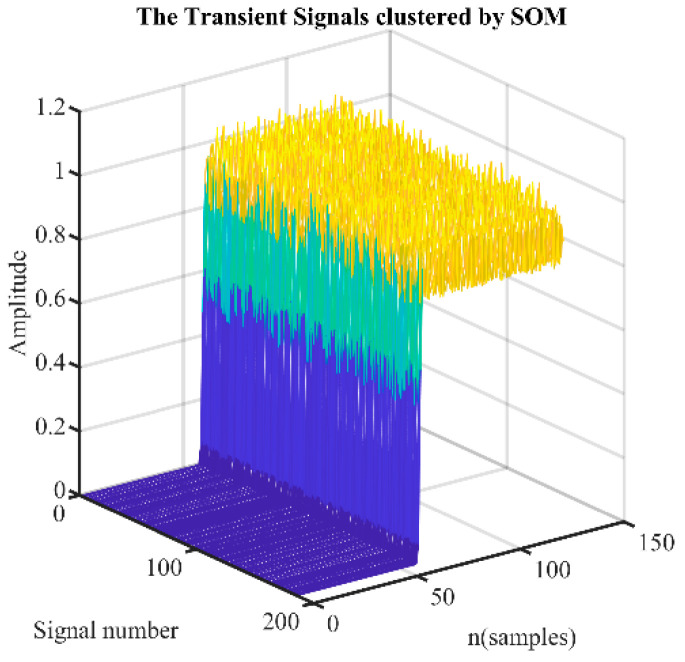
The transient signals clustered by SOM.

When multiple UAVs appear in the detection area, due to the differences in their radios and locations, the statistical characteristics can be used for the SOM clustering of UAV transient signals. It includes features such as variance ($\sigma^{2}$), crest factor (C), pulse factor (I), clearance factor ($C_{e}$), kurtosis factor (K), shape factor ($S_{f}$), and skewness (S).

(8) $$\sigma^{2}=\frac{1}{N}\sum_{i=1}^{N}{\left(x_{i}-\bar{x}\right)}^{2}$$

(9) $$C=\frac{\max\left\{\left|x_{i}\right|\right\}}{\sqrt{\frac{1}{N}\sum_{i=1}^{N}{x_{i}}^{2}}}$$

(10) $$I=\frac{\max\left\{\left|x_{i}\right|\right\}}{\frac{1}{N}\sum_{i=1}^{N}\left|x_{i}\right|}$$

(11) $$C_{e}=\frac{\max\left\{\left|x_{i}\right|\right\}}{{\left(\frac{1}{N}\sum_{i=1}^{N}\sqrt{\left|x_{i}\right|}\right)}^{2}}$$

(12) $$K=\frac{\frac{1}{N}\sum_{i=1}^{N}{\left(x_{i}-\bar{x}\right)}^{4}}{\sigma^{4}}$$

(13) $$S_{f}=\frac{\sqrt{\frac{1}{N}\sum_{i=1}^{N}{x_{i}}^{2}}}{\frac{1}{N}\sum_{i=1}^{N}\left|x_{i}\right|}$$

(14) $$S=\frac{\frac{1}{N}\sum_{i=1}^{N}{\left(x_{i}-\bar{x}\right)}^{3}}{\sigma^{3}}$$

where the $x_{i}$ is the vector of the transient signal and N is the length of the vector. The clustering signals’ joint identification algorithm identifies telemetry radios by using the signals divided in the same cluster by SOM. See Figure 8.

The final score of this algorithm is based on the plurality voting of the results. This voting process achieves better accuracy compared with a single signal identification. The probability of the signal label is defined as follows:

(15) $$p(i)=\frac{K(i)}{S_{cluter}}\;\;\;\;\;\;$$

where K(i) denotes the number of transient signals, in which label is i identified by the CNN model and $S_{cluter}$ represents the total number of transient signals in the cluster.

The UAV clustering signals joint identification algorithm is given by

(16) $$Lable_{predict}=arg\;max(p(i))$$

## 5. Experiments and Results Analysis

### 5.1. Experimental Setup and Data Capture

During the indoor experiments, RF signals are captured from 20 UAV telemetry radios (Quansheng Electronics Company, Dongguan, China) with the same model from the same manufacturers. All the radios transmit signals in the 915 MHz frequency band. This is important to investigate the confusion that would arise when a UAV RF footprint identification system attempts to distinguish UAV telemetry radios of the same model.

To build the UAV RF signal-capturing environment, we need to select a UAV flight control platform to connect to the telemetry radio first. Pixhawk/APM (Quansheng Electronics Company, Dongguan, China) is the hardware and software platform widely used in the field of open-source UAV research and application. In this paper, the UAV RF signal-capturing environment is based on the Pixhawk/APM platform combined with the SiK radio [31].

**Figure 9:**
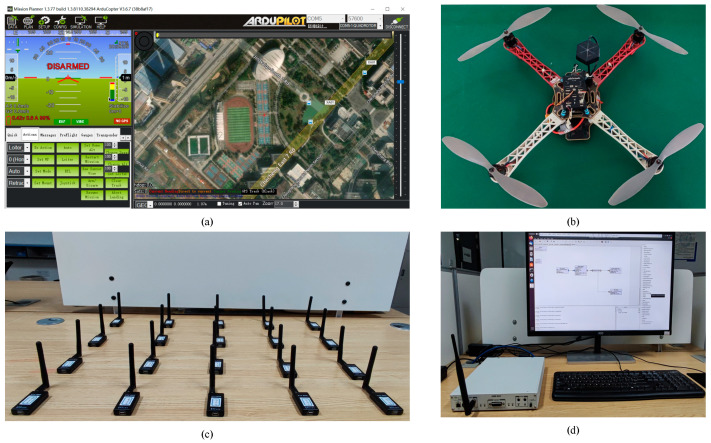
UAV RF signal capturing environment. (**a**) GCS-Mission Planner, (**b**) UAV platform with Pixhawk/APM, (**c**) SiK telemetry radios (Quansheng Electronics Company, Dongguan, China), and (**d**) USRP X310 with GNU Radio.

The USRP X310 equipped with a UBX 160 daughterboard (Ettus Research, Santa Clara, CA, USA) is applied to capture wireless signals in the experiment environment. It is a high-performance scalable software-defined radio (SDR) platform with up to 160 MHz of baseband bandwidth and it covers frequencies between 10 MHz–6 GHz [32]. The GNU Radio, which can be used with readily available low-cost external RF hardware, is an open-source development toolkit. The signal flow chart for capturing signals is designed in the GNU Radio software 3.8. The sampling center frequency is 921 MHz and the sampling rate is 15 MS/s. After the sampling command is executed, binary IQ data from the UAV telemetry radio signal will be saved to a local file. Each captured signal consists of millions of sampling points. The size of the file depends on the time of signal sampling. Figure 9 shows the indoor experimental scenarios.

### 5.2. Performance of Transient Signal Detection

The close sampled signals can be regarded as a noiseless waveform in the experiment. It is important to evaluate the transient signal detection performance of the EC−α algorithm used in our experiments at different SNR levels. To achieve these goals, AWGNs are added to raw signals in this experiment. Then, the performance of the EC−α method is assessed in terms of detection accuracy.

A noisy signal is randomly selected from 1200 training samples and the starting point detection accuracy of the EC−α algorithm is evaluated at different SNRs. The experiment is repeated 100 times for each performance evaluation and the average results are calculated as the final performance evaluation result.

**Figure 10:**
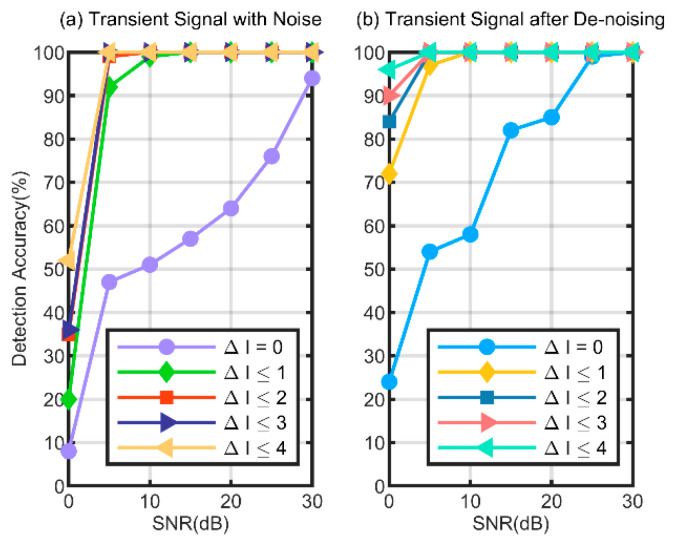
Starting point detection accuracy of different error thresholds.

In the assessment, the absolute error metric is used as follows:

(17) $$\Delta I=\left|I_{0}-I_{d}\right|$$

where $I_{d}$ is the estimated start of the transient signals and $I_{0}$ is the actual start of the transient signals. The detection performance of the starting point is shown in Figure 10 at an SNR ranging from 0 dB to 30 dB. The EC−α algorithm is used directly to detect transient signals with different levels of noise, as shown in Figure 10a. The noisy signal is preprocessed by the wavelet de-noising method first; then, transient signals are processed using the EC−α algorithm, as shown in Figure 10b.

As illustrated in Figure 10a, the accuracy of starting point detection improves with higher SNR. Due to the influence of noise, there will be a certain error between the estimated position and the actual position. At SNR = 0 db, 52% of the absolute errors ΔI do not exceed four sampling points and only 8% of experiments are ΔI =  0. When SNR reaches 10 dB, 99% of ΔI is not greater than one sampling point. It is apparent from Figure 10b that after de-nosing the detection accuracy has been greatly improved. Compared with Figure 10a, 96% of the transient signal detection error does not exceed four sampling points at SNR = 0 db. When SNR reaches 5 dB, 97% of ΔI is not greater than one sampling point. When SNR is increased to 15 dB, 82% of the transient signal detection error reaches 0.

### 5.3. CNN-Based Raw I/Q Data

#### 5.3.1. Performance of the Baseline CNN on the Validation Dataset

We use 20 different telemetry radios of the same model to validate the performance of our proposed baseline CNN. For each device, we capture about 1.25K transient signal samples as the dataset. During the experiments, we use 60% of the dataset for training, 20% of the dataset for validation, and 20% of the dataset for testing. The CNN model is trained using the raw I/Q data of the transient signals.

Generally, configuration parameters of a CNN, such as parameters of convolution lays and max pooling lays, etc., all affect the network performance and are hard to optimize. Hence, we adopt the grid search for tuning configuration parameters of the baseline CNN. The parameters of the convolutional layers and max pooling lays are shown in Table 2. Dropout operators are used on the fully connected layer to prevent overfitting.

**Figure 11:**
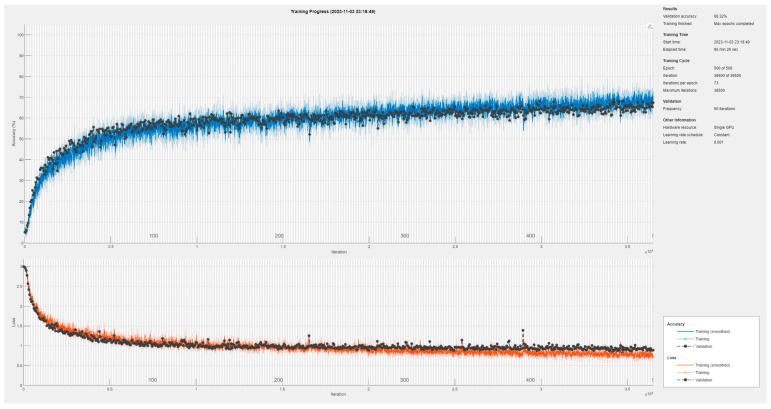
Training progress of the baseline CNN model.

We determine the best hyper-parameters based on the identification accuracy of the validation dataset. Our training is conducted by optimizing the cross-entropy loss function using an Adam solver. The batch size is 256 and the initial learning rate is 0.001. The whole training process repeats until the Max Epochs is finished and the best validation model is stored for evaluation. All our network models are trained and tested running on MATLAB R2022 with an RTX A5000 GPU (NVIDIA, Santa Clara, CA, USA). The baseline CNN training progress is shown in Figure 11. After 150 epochs of training, the identification accuracy has reached 60%. In the last 350 epochs of training, the improvement in identification accuracy is very limited. We only obtained 68.32% accuracy on the validation dataset in the SNR range from 0 dB to 30 dB. More specifically, in the case of low SNR, some useful information is eliminated together with the noise, resulting in a sharp decrease in identification performance. Obviously, the identification accuracy of the baseline CNN model is unsatisfactory.

#### 5.3.2. CNN Depth Impact Analysis

Network depth is also one of the important factors affecting CNN’s performance. As we know, deeper CNNs in image processing can extract more feature relationships from the original dataset with the same number of parameters. To improve the identification performance of our CNN network, we try to build deeper networks to learn more complex features of raw I/Q data from transient signals. We conduct experiments based on the baseline CNN and stack different numbers of feature extraction blocks to evaluate the impact of network depths.

Identification accuracies for CNNs with different depths are illustrated in Table 3. In contrast to the studies in image recognition, increasing the number of convolutional layers shows no improvement in UAV transient signals identification here.

The training progress of the CNN Model with four feature extraction blocks indicates that the CNN begins to be more overfitting with more convolutional layers used. As the number of training epochs increases, the accuracy of the training dataset can continue to improve. However, after 120 training epochs, the accuracy of the validation dataset did not improve and the loss gradually increased. In a word, the RF footprint identification accuracy of UAV telemetry radios is not limited by CNN network depth in our experiment. Simply increasing the depth of a CNN model cannot always improve signal identification performance.

### 5.4. SOM Clustering Accuracy Analysis

To further improve the identification performance, we propose a joint identification optimized method using SOM clustering signals. First, it is necessary to evaluate the clustering accuracy of the SOM algorithm for transient signals from different UAV telemetry radios.

**Figure 12:**
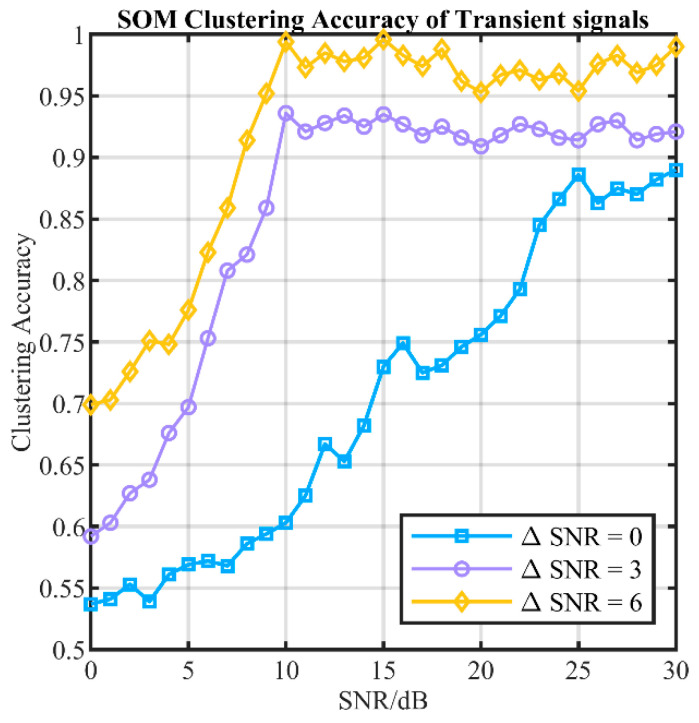
SOM clustering accuracy of UAV transient signals.

In our experiments, two devices are randomly selected from 20 telemetry radios each time and then 260 transient signals are randomly selected from the test dataset of each device for random mixing. Then, the SOM algorithm is used to perform clustering and the clustering accuracy of the top three clusters is calculated. To analyze the environment under different SNR conditions, the software generates AWGN to simulate channel noise at the desired SNR, making the noisy waveform. We test the clustering accuracy under three conditions where the SNR difference in two radios is 0 dB, 3 dB, and 6 dB. The test under each condition is repeated 50 times to obtain the average accuracy. The test results are shown in Figure 12. The clustering accuracy is related to the SNR of the reference signal and the SNR difference in the two devices. Especially when the SNR difference between the two devices is larger, the accuracy of clustering will be higher. When the SNR difference is 6 dB and the reference signal SNR is greater than 10 dB, the clustering accuracy is above 95%. When the SNR difference is 3 dB and the reference signal SNR is greater than 10 dB, the clustering accuracy is above 90%.

### 5.5. Performance of the Baseline CNN on the Test Dataset

**Figure 13:**
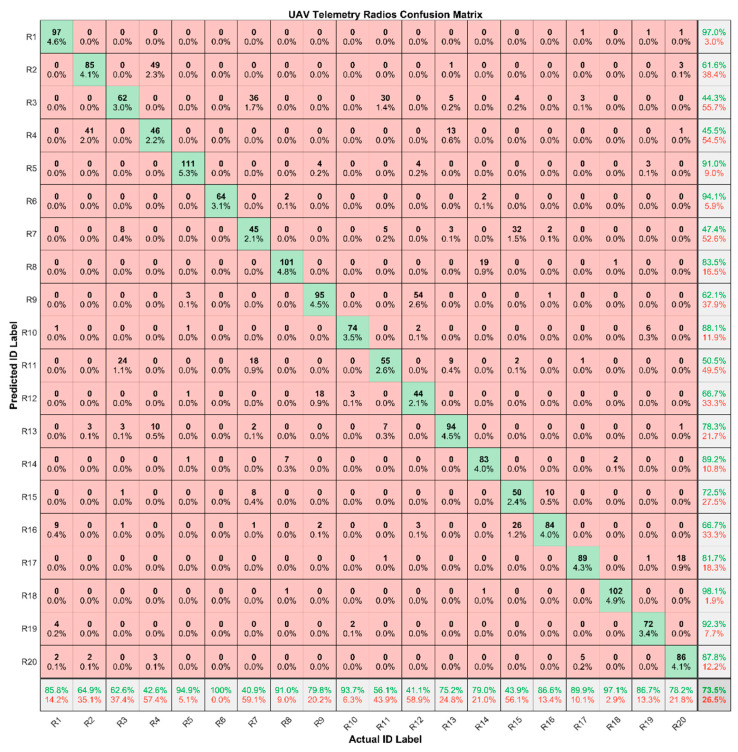
The Confusion Matrix of the baseline CNN on the test dataset.

We use the trained baseline CNN model to identify the test dataset clustered by SOM. The average identification accuracy on the test dataset is 73.5% at 20 dB. The Confusion Matrix of the baseline CNN on the test dataset is shown in Figure 13. The identification performance of the baseline CNN model for different radios varies greatly. The worst identification accuracy of a single device is 40.9% and the best one is 100%. The proportion of correctly identified signals for each radio also varies. For most radios, the number of signals in the cluster correctly identified is the majority.

### 5.6. Performance of Clustering Signals Jointly Identification

To verify the performance of the CSJI algorithm, we identify the signals clustered at different SNRs in the previous section. At the same time, we extract several features such as amplitude and phase features from the raw I/Q samples and build a rich set of features to train the SVM and KNN. We use SVM and KNN to compare and analyze the identification performance of different algorithms on the test dataset at different SNRs.

**Figure 14:**
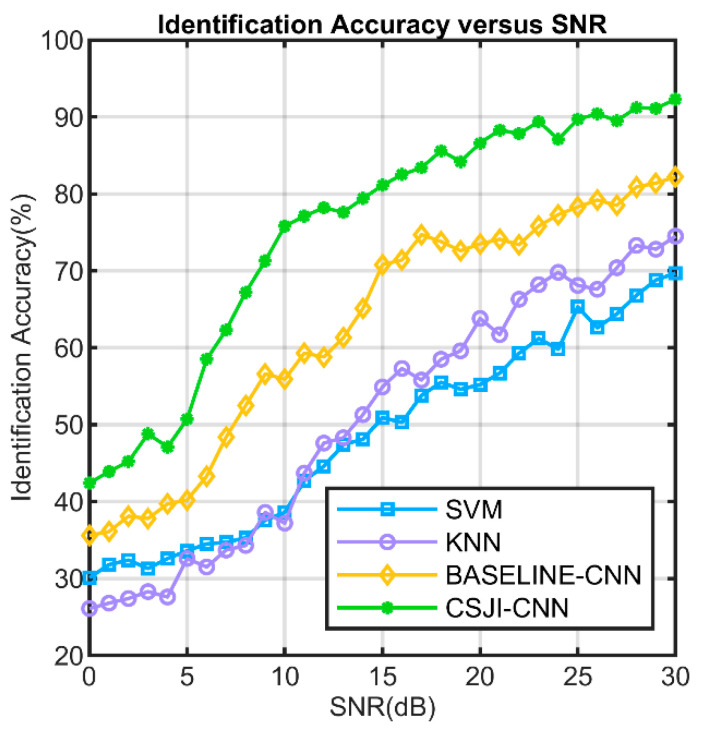
The identification accuracy versus SNR for different algorithms.

Figure 14 shows the identification accuracy versus SNR for SVM, KNN, baseline CNN, and CSJI-CNN. It is obvious that the identification performance of the four algorithms improves as the SNR increases. As the SNR decreases, the features in the transient signals are lost in the noise. Under the same SNR conditions, CNN has a higher identification accuracy than SVM and KNN because the CNN network can extract more radio frequency fingerprint features from the raw I/Q data. However, the baseline CNN’s identification performance of 20 devices of the same model is still very limited and can only reach 82.2% at 30 dB. Since the same type of UAV telemetry radio uses the same type of hardware, the RF fingerprint has a stronger similarity. The CNN model based on raw I/Q data features has limited ability to identify the same type of UAV telemetry radio. Through the CSJI algorithm, the performance can be effectively improved to 92.3% at 30 dB. In comparison, the identification accuracy of SVM and KNN is 69.7% and 74.5%, respectively, at the same SNR condition.

### 5.7. Performance in Outdoor Experiments

**Figure 15:**
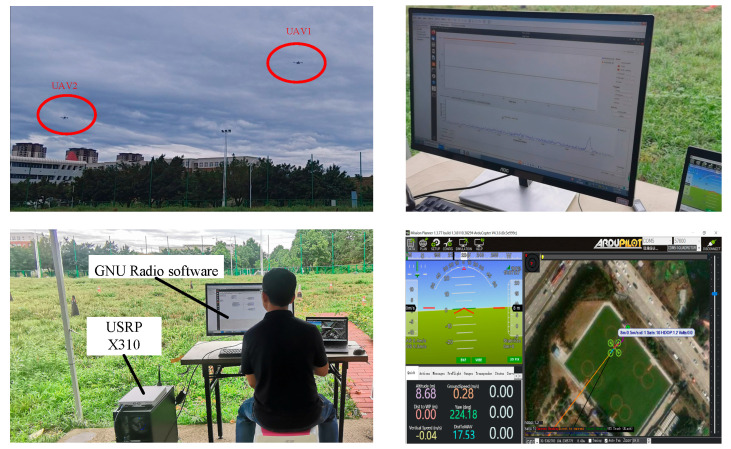
The outdoor experimental scenarios.

In the outdoor test, we use two UAVs to fly in the same area, and the distance between UAVs and GCS is about 150 m. In the flight test, each UAV is equipped with a pair of telemetry radios selected from 20 training devices. We repeated the experiment multiple times until 20 radios had been used. To avoid the influence of the GCS radios, we disconnect the GCS radios and switch the UAVs to manual mode before sampling data and then the raw I/Q data of the telemetry radio are sampled. The estimated SNR of the signals in this scenario is around 25 dB. Figure 15 shows the outdoor experimental scenarios.

After processing the sampled data according to the procedure in Figure 2, through the CNN model and CSJI algorithm, the identification accuracy in the entire flight test is 76.1%, which declined by 13.6% compared to the accuracy of 89.7% at 25 dB in the indoor test environment. We observe that identification accuracy decreases as communication channels have an important impact on the extraction of RF fingerprint features. This is because our CNN model is trained using raw I/Q data, which are collected in the indoor environment. Multipath reflection and fading have a considerable impact on received I/Q samples when the test is conducted outdoors. The impact of the channel becomes even more severe when the training and testing occur on different days. To further improve the performance in outdoor scenarios, we need to generate datasets under various channel conditions to train the demanded CNN in future work.

## 6. Conclusions

This paper has demonstrated a UAV RF fingerprinting identification approach based on a CNN model trained by raw I/Q data of the transient signals. Furthermore, we propose the CSJI algorithm to further improve the identification performance. It has been proven by the indoor and outdoor tests that our approach can identify features embedded in the raw I/Q data of UAV telemetry radios. Compared with SVM and KNN, the CSJI algorithm improves the identification accuracy of UAV telemetry signals by 22.6% and 17.8% at 30 dB, respectively. In future work, we will focus on increasing the robustness of the CNN model to enhance the identification accuracy in low SNR scenarios. The outdoor time-varying channel will be considered and the number of UAV telemetry radios will be increased.

## Figures and Tables

**Table 1:** Related work on UAV RF-based detection and identification.

Literature	Identification Targets	Pre-Processed and Detection Method	Signal Features	Identification Algorithms	Number of the Test Device	Accuracy
[22]	UAV controller signals	a single level Haar wavelet decomposition/a statistical changepoint detection algorithm	scattergrams from the steady state of the RF signals	SqueezeNet	10 different UAV controllers	98.9% at 10 dB SNR
[23]	UAV controller signals	Markov models-based naïve Bayes decision mechanism	the three most significant features of the fifteen statistical features extracted from the energy transients	KNN classifier	15 different types of UAV controllers	98.13% at 25 dB SNR
[24]	UAV WiFi signals	discrete Fourier transform	spectra of the UAV RF signals	deep neural networks	3 different UAV controllers	46.8% for10-classes
[25]	UAV controller signals	Empirical mode decomposition	slope, kurtosis, and skewness of the signals	BPNN	a UAV controller	greater than 82% within a distance of 3 km
[26]	UAV downlink video transmission signals	data augmentation and spectrogram segmentation	subspectrogram	lightweight ResNet	5 UAV types	accuracy of 95% When SINR ≥ 15 dB
[20]	UAV controller signals	GoF spectrum sensing	spectrogram image	YOLO-lite DRNN	nine commercial UAVs	F1-score 97% at −3 dB

**Table 2:** The configuration parameters of the baseline CNN.

Layer	Feature Extraction Block Number	Parameters	Activation
Input	-	2 × 128	-
Convolution	Feature Extraction Block 1	1 × 7 × 64	-
Convolution		1 × 5 × 64	RELU
Max Pooling		3 × 3	-
Convolution	Feature Extraction Block 2	1 × 7 × 64	-
Convolution		1 × 5 × 64	RELU
Max Pooling		3 × 3	-
Convolution	Feature Extraction Block 3	1 × 7 × 64	-
Convolution		1 × 5 × 64	RELU
Max Pooling		3 × 3	-
Dropout	-	0.6	-
Fully Connected	-	64	-
Dropout	-	0.6	-
Fully Connected	-	20	Softmax

**Table 3:** CNN performance with different network depths.

The Number of Feature Extraction Blocks	The Number of Convolutional Layers	Training Time	Best Validation Accuracy
2	4	69 min 28 s	57.75%
3	6	95 min 26 s	68.32%
4	8	112 min 56 s	63.13%

## Data Availability

Dataset available on request from the authors.

## Abbreviations

The following abbreviations are used in this manuscript:

UAVs	Unmanned Aerial Vehicles
BVLOS	Beyond Visual Line of Sight
RF	Radio Frequency
SNR	Signal to Noise Ratios
CNN	Convolutional Neural Network
SOM	Self-Organizing Map
CSJI	Clustering Signals Joint Identification
USRP	Universal Software Radio Peripheral
KNN	K-Nearest Neighbor
SVM	Support Vector Machine
GCS	Ground Control Stations
AWGN	Additive White Gaussian Noise
ReLU	Rectified Linear Unit
